# Strong ion difference and arterial bicarbonate concentration as cornerstones of the impact of fluid therapy on acid-base balance

**DOI:** 10.1186/cc12316

**Published:** 2013-03-19

**Authors:** D Ottolina, M Ferrari, L Zazzeron, E Scotti, M Stanziano, C Rovati, C Marenghi, L Gattinoni, P Caironi

**Affiliations:** 1 Fondazione IRCCS Ca' Granda - Ospedale Maggiore Policlinico Università degli Studi di Milano, Milan, Italy

## Introduction

The biochemical characteristics of infused fluids may be important in regulating acid-base balance, by modifying plasmatic volume and strong ion difference. *In vitro *and animal studies [1,2] have shown that volume and strong ion difference of infused fluids (SIDin) as well as the arterial baseline bicarbonate concentration (HCO_3_-a) influence acid-base variations. Our aim was to verify these changes in critically ill patients after surgery.

## Methods

An electronic-dedicated database was created to retrospectively collect volume, type of fluids infused and plasmatic acid-base balance variations in postoperative ICU patients from admission to 9:00 am of the day after. SIDin was calculated as the average SID of all fluids infused during the whole study period (crystalloids, colloids and blood products). Arterial base excess variation (ΔBEa) was computed as the difference between values at 9:00 am on the day after and those at entry. We report data from all patients admitted in 2006 and 2007 (650 patients).

## Results

Nine patients not receiving intravenous infusions were excluded. The remaining population was divided into three groups according to SIDin distribution (Group 1, 18 ± 12; Group 2, 47 ± 6; Group 3, 55 ± 0 mEq/l). We observed a progressive increment in ΔBEa between the groups (1.1 ± 2.0 vs. 2.8 ± 2.9 vs. 3.0 ± 2.8 mmol/l, *P <*0.001). We further subdivided each group by the median value of baseline HCO_3_-a (24.3 (22.3 to 26.1) mmol/l) and we analyzed the ΔBEa: we observed a greater increase in patients with lower baseline HCO_3_-a (Group 1, 1.8 ± 2.9 vs. 0.2 ± 2.6, mmol/l, *P <*0.001; Group 2, 4.0 ± 2.7 vs. 1.5 ± 2.6, mmol/l, *P <*0.001; Group 3, 4.4 ± 2.8 vs. 1.7 ± 2.0 mmol/l, *P <*0.001), as compared with those with higher baseline levels. When the study population was divided into quartiles of the difference between SIDin and HCO_3_-a, ΔBEa appeared to increase with the rise of such difference (*P <*0.001).

## Conclusion

SIDin affects the acid-base status *per se *and in relationship with HCO_3_-a. We verified this hypothesis in critically ill patients, highlighting the importance of the difference between SIDin and HCO_3_-a, which better describes and predicts the acid-base modifications to fluid therapy.
